# A Retrospective Analysis on Clinical Practice-Based Approaches Using Zolpidem and Lorazepam in Disorders of Consciousness

**DOI:** 10.3390/brainsci11060726

**Published:** 2021-05-29

**Authors:** Bei Zhang, Katherine O’Brien, William Won, Sheng Li

**Affiliations:** 1 Department of Physical Medicine and Rehabilitation, McGovern Medical School, University of Texas Health Science Center at Houston, Houston, TX 77030, USA; Bei.Zhang@uth.tmc.edu (B.Z.); William.W.Won@uth.tmc.edu (W.W.); 2 TIRR Disorders of Consciousness Program, TIRR Memorial Hermann Hospital, Houston, TX 77030, USA; 3 H. Ben Taub Department of Physical Medicine and Rehabilitation, Baylor College of Medicine, Houston, TX 77030, USA

**Keywords:** disorders of consciousness, zolpidem, lorazepam, traumatic brain injury, anoxic brain injury

## Abstract

This is a retrospective study to investigate the results of using zolpidem and lorazepam in persons with disorders of consciousness (DoC) and to provide practical information for clinical application and further studies. The cohort included 146 patients (11 hemorrhagic stroke, 87 traumatic brain injury (TBI), 48 anoxic brain injury (ABI)) admitted to a specialized DoC rehabilitation program. A positive trial indicated a patient responded to either zolpidem or lorazepam with prominent functional improvements necessitating routine use of the medication. Non-responders had equivocal or negative (i.e., went to sleep) responses. Eleven patients with a stroke who had either medication were all non-responders. Of the remaining 135 patients, 95 received at least one medication trial. The overall positive rate was 11.6% (11/95), with 6.3% (5/79) for zolpidem and 14.0% (6/43) for lorazepam. Among TBI patients, the positive rate of the zolpidem trial (10.2%, 5/49) was slightly higher than that of the lorazepam trial (6.9%, 2/29; *p* > 0.05). Among ABI patients, the positive rate of the lorazepam trial (28.6%, 4/14) was significantly higher than that of the zolpidem trial (0%, 0/30; *p* = 0.007). Following a positive trial, most patients were continued on the medications on a regular basis before eventual discontinuation. Our results suggested the etiology of DoC, considering traumatic vs. anoxic injuries, may serve in guiding the clinical application of these medications in the treatment of DoC and in future prospective studies. We advocate for screening all patients with DoC using zolpidem and/or lorazepam.

## 1. Introduction

Disorders of consciousness (DoC), including coma, unresponsive wakefulness syndrome/vegetative state (UWS/VS), and minimally conscious state (MCS), are possible consequences following a severe brain injury [1]. The only pharmacological treatment that has shown benefit in a randomized controlled trial is amantadine, which accelerated functional recovery in acute-to-subacute traumatic UWS/VS and MCS [2,3]. Since the first report of magic awakening from a “vegetative” state after taking zolpidem in 2000 [4], the paradoxical responses to the hypnotic medications in DoC have drawn much attention in both clinical and research fields over the past two decades. The most widely reported agent is zolpidem. Numerous case reports have been published on its effectiveness on arousal, physical abilities, verbal communications, and cognitive functions [5,6,7,8,9]. Three major prospective clinical studies revealed that 6.7% (1/15) [10], 4.8% (4/83) [11], and 3.3% (2/60) [12] patients responded positively with improvement in the diagnostic categories (e.g., improved from a UWS/VS to an MCS) after administering zolpidem. Benzodiazepines are less well-known as an “awakening” medication, with very few case reports on its application in persons with DoC [13]. One patient, presumably having DoC, improved significantly in cognition, language, and mobility with 1 mg of lorazepam. It was believed that the improvements were attributed to the relief of underlying catatonia [13]. Nonetheless, paradoxical disinhibition/excitation to benzodiazepines is well documented in various healthy subjects and patients with substance abuse and psychiatric disorders, causing talkativeness, restlessness, aggressive behaviors, etc. [14].

Although the clinical studies did not show its general efficacy, the use of zolpidem has been proven effective in a select subset of individuals with DoC. Its therapeutic effect could be so prominent or dramatic that, as entitled in a New York Times Magazine article, “wakes the near-dead” [15]. This could potentially be life-changing for a patient with DoC. Efforts have been made to determine predictors for the responders. One study showed the responders were the ones with coup-contrecoup injuries and space-occupying lesions rather than those with brainstem injuries [16]. Others suggested brain areas with impaired function may be more revealing than demographic or mechanistic factors [11,17]. To date, no phenotypical characteristics were identified to guide the clinical application of these medications [17]. However, it was suggested that it is relatively feasible to screen DoC patients with a single-dose zolpidem trial, considering the potential benefits significantly outweigh the risks [10]; no clear recommendation was made for lorazepam. Endorsing this strategy in our specialized DoC rehabilitation program, the attempt was made to optimize the possibilities of recovery that one may benefit from either zolpidem or lorazepam. We retrospectively reviewed the zolpidem and lorazepam trials over a period of 5 years, hoping to provide clinically practical information for the use of these medications in treating persons with DoC and to facilitate further studies.

## 2. Materials and Methods

The cohort included 146 patients who were admitted to a DoC rehabilitation program from 1 January 2014 to 31 October 2018, as previously described [18,19]. The charts were reviewed, and relevant information was extracted from the electronic medical record system. Basic demographics included age at the time of injury, sex, etiology, and time since injury on admission. The results of the zolpidem and lorazepam trials were analyzed, respectively. A positive response indicated a patient became more aroused and/or showed significant functional improvements. This was observed, typically started 30 min after administering the medication, in 2 consecutive therapy sessions, physical (usually 60 min) and/or occupational (usually 60 min) and/or speech therapy (usually 30 min), by the primary treating therapists, with a 0–30 minutes’ break in between sessions. Experienced neuropsychologists specialized in assessing conscious states, and the primary physiatrists were actively following. Information was also collected from other DoC care team members (e.g., nursing) and families across the day. The Coma Recovery Scale—Revised (CRS-R) is performed on a regular basis but not always on the days of the trials. The immediate evaluation by their primary therapists in various treating environments with optimal stimulation was prioritized as it has the best chance of showing a patient’s functional changes (eliminating the limitations on one’s potential under a testing scheme). A positive response necessitated administering the medication on a daily basis to facilitate functionality and rehabilitation progress, based on the consensus reached among the professionals in the DoC care team. An equivocal response indicated a patient did not show any arousal or functional improvements but also did not become drowsy or fall asleep. A negative response indicated a patient became drowsier or fell asleep following the administration of the medication. Almost all trials were conducted open-label; a 4-day blind trial series was conducted in 3 patients to confirm previous trial results due to inconsistent observations from the team members and/or families. Those who had a positive trial were categorized as “responders”; otherwise, as “non-responders.” Subsequently, attempts were made to summarize the titration and management of the trial medications, e.g., when it was scheduled, details of functional improvements, whether it was discontinued eventually, whether functional gains sustained or subsided or went beyond, what the long-term outcomes were among the responders. The dosage and titration of amantadine, the consciousness level around the period based on CRS-R were also reviewed.

Data were collected and analyzed using Microsoft Office Excel 2007. Numerical variables were presented by their distributions. Categorical variables were presented as numbers or percentages. Due to the small sample size of the patient population and trial sessions/results, concerning it becoming a potential confounder, all patients with an isolated stroke as the etiology were excluded from subsequent analysis. Fisher’s exact test was used to analyze the differences in the proportion of responders based on different medications used and different etiologies. Statistical significance was set at *p* < 0.05.

## 3. Results

### 3.1. Overall Results of the Zolpidem and Lorazepam Trials

The cohort consisted of 146 brain injury patients, including 11 patients with a hemorrhagic stroke, 87 patients with a traumatic brain injury (TBI), and 48 patients with an anoxic brain injury (ABI). Stroke patients who had either medication were all non-responders (0/3 for the zolpidem trial and 0/4 for the lorazepam trial). After excluding stroke patients, the new cohort consisted of 135 patients. The basic demographic information is presented in Table 1. Overall, 95 patients received at least one trial (68 received either zolpidem or lorazepam; 27 received both), while 40 patients received neither. The overall positive rate was 11.6% (11/95), among which the positive rate for the zolpidem trial was 6.3% (5/79) and for the lorazepam trial was 14.0% (6/43; *p* > 0.05). No adverse effects or events were reported.

Details about the trials and the demographics of the responders and the non-responders to the zolpidem and lorazepam trials are presented in Table 2. The number of patients who received a zolpidem trial was proportionally similar between TBI and ABI patients (56.3% vs. 62.5%, respectively). The same was found for the lorazepam trial (33.3% vs. 29.2%, respectively). Among TBI patients, the positive rate of the zolpidem trial was slightly higher but not statistically different from that of the lorazepam trial (10.2% vs. 6.9%, *p* > 0.05). In contrast, among ABI patients, the positive rate of the lorazepam trial was significantly higher than that of the zolpidem trial (28.6% vs. 0%, *p* = 0.007). In general, the responders were younger than the non-responders in both zolpidem and lorazepam trials among TBI and ABI patients. Other characteristics were largely indistinguishable among the responders. Among those 27 patients who had both trials, none responded positively to both medications in the limited trials performed in varying sequences. Interestingly, four lorazepam responders had at least one prior negative or equivocal zolpidem trial.

### 3.2. Following a Positive Trial

The demographics, trial information, and long-term outcomes of the five zolpidem and the six lorazepam responders were summarized in Table 3 and Table 4, respectively. Clinical management and trajectories varied significantly based on patients’ clinical conditions and responses to either medication. Most patients were started on the trial medications on a daily basis or twice daily in the early morning and at around noon to facilitate arousal and participation in therapies. It was noted that before scheduling the medications, multiple trials with different dosages, frequencies, at different times during daytime, and on different days were conducted to confirm the response and to explore for an optimal regimen. After scheduling the medications, close monitoring with active titration was also performed. In at least 81.8% of the responders (9/11), these medications were discontinued eventually, most commonly because the patients had improved to a functional level not requiring it, while in some cases, they were considered not helpful anymore or discontinued by physicians at other facilities. Although the responders tended to have a higher likelihood of achieving emergence (9/11, 81.8% vs. 39/84, 46.4%, *p* = 0.051), it has to be noted that 4 of them, by the time of responding to zolpidem or lorazepam, had been found to have emerged (Case 3, 9, 10, 11). Nevertheless, 5 of them came to clear emergence shortly after positive trials from MCS (Case 1, 2) or marginal emergence * (MCS/eMCS; Case 5, 7, 8). Long-term (over 6 months) outcomes also varied significantly among the responders. Relatively better outcomes were seen in Case 1 (MCS → eMCS), 3 (eMCS → eMCS), 7 (MCS/eMCS → eMCS), and 8 (MCS → eMCS), while relatively poorer outcomes in Case 4 (UWS/VS → UWS/VS; complicated by medical conditions around the trial time), 5 (MCS/eMCS → eMCS), 6 (MCS → MCS), 10 (eMCS → eMCS), and 11 (eMCS → eMCS). It may be correlated with the magnitude of the trial response that led to an improvement in clinical diagnostic categories. (* The idea of “marginal emergence” is to describe an intuitive transitional period when a patient becomes close to eMCS but not yet reflecting on the standardized testing (Case 5 and 8) or achieves one-time eMCS on standardized testing but not consistently reflecting on other times and on clinical observation (maybe related to instability of the neural network during natural recovery or other confounding medical conditions; Case 7). It is used here to provide the readers a better sense of a patient’s consciousness level. In other words, simply using “MCS” or “eMCS” may present an impression to either underestimate or overestimate their consciousness levels at that time.).

### 3.3. Following an Equivocal or a Negative Trial

In many patients, at least one repeated trial was conducted in the next day or the next few days using the same or the other agent. Repeated trials were also seen weeks or months apart depending on a patient’s condition and the admission phases. Due to the limitation of the retrospective study in an uncontrolled setting, no specific clinical management pattern could be summarized. As shown above, despite the trial results, a significant number of DoC patients were able to achieve emergence.

## 4. Discussion

In this retrospective study, several intriguing findings were revealed, e.g., a higher response rate to zolpidem in TBI-related DoC patients and a higher response rate to lorazepam in ABI-related DoC patients. This etiology-related response pattern was not recognized before. These findings may help guide treatment for persons with DoC clinically and shed light on further studies.

The overall positive response rate to zolpidem was 6.3%, similar to previous reports [10,11,12]. Interestingly, we observed an approximately 10% positive response rate to zolpidem in TBI-related DoC patients. By extracting data from previous prospective studies, similar results were shown (12.5%, 1/8 in Whyte 2009; 11.1%, 7/63 in Whyte 2014; 25.8%, 8/31 in Thonnard 2013) [10,11,12], which adds validity to our current results. In ABI-related DoC, similarly, only a few patients had a positive response to zolpidem (0%, 0/5 in Whyte 2009; 10.0%, 1/10 in Whyte 2014; 11.1%, 2/18 in Thonnard 2013) [10,11,12]. However, surprisingly, nearly 30% of the ABI-related DoC patients responded to lorazepam in our cohort. No similar studies could be found to make the comparison. None of the patients with an isolated stroke responded to zolpidem in the above prospective studies as well as in our cohort.

The differences in the pharmacodynamics of these two medications may help explain the differences in response in different etiologies. Over the years, the common neural substrate for DoC, a mesocircuit-frontoparietal model underlying the graded return of responsiveness, has been better elucidated with advances in neuroimaging and electrophysiological techniques [2]. Within the proposed circuit, loss of inhibition of the globus pallidus interna (GPi) produces active inhibition of the central thalamus, subsequently causing functional suppression of the anterior forebrain [2,11]. Zolpidem selectively binds to the α1 subunit of the GABA_A_ receptors, which is highly concentrated in the GPi, thus inhibiting the activity of the GPi and reversing the suppression of the higher-order frontal cortices [11,20]. Different from zolpidem, lorazepam is non-selective and binds to a modulatory site on the GABA_A_ receptors [20]. It may exert effects on a wider neural network containing GABA_A_ receptors composed of other types of α subunit (e.g., α2-, α3-, α5-containing receptors bind benzodiazepines with high affinity) [20]. Studies have shown that GABA_A_ receptor subtypes have distinct anatomical localizations and mediate diverse functions [20,21]. In a very interesting case with catatonia, lorazepam helped with all motoric and neuropsychiatric symptoms but not mutism; adding zolpidem resulted in a dramatic improvement in spontaneous verbalization [22]. There are other reports supporting specific effects of zolpidem on the language network, including improving the behavioral aspects of speech (initiation and motivation) [22], increasing regional cerebral blood flow [5,23], and counteracting the dynamic diaschisis in the language network [23]. These suggested the two medications may exert distinct effects on different neural networks, both of which may potentially be beneficial in the treatment of DoC from different etiologies. However, their specific mechanisms warrant further investigation.

It is worth noting the high positive response rate to lorazepam in ABI-related DoC patients. Among the responders, many of them were suspected of having catatonic features. The trials were conducted in similar proportions of the TBI-related and ABI-related DoC patients (Table 2), which helps partially offset potential patient selection bias. Catatonia may mimic the presentations of DoC (e.g., mutism, immobility, posturing, etc.) [1,13]. Lorazepam could be used for diagnostic and therapeutic purposes [24]. Currently, it is unclear if ABI is related to a higher incidence of catatonia and how catatonia or catatonic features interplays with DoC.

### 4.1. Insights on Clinical Practice and Future Studies

The zolpidem and lorazepam trials are relatively safe and inexpensive to perform [25,26]. When it is effective, at least at an individual level, it may exert a life-changing impact on the person’s functionality, recovery trajectory, rehabilitation resources one may receive in the long run, etc.; as seen in Case 1, 3, 7, 8. Considering the very limited treatment options currently available for persons with DoC, we would like to advocate for screening all DoC patients with zolpidem and/or lorazepam when medically stable, as previously suggested [1,2,10]. This could be performed in various settings (e.g., acute care units, long-term acute care hospitals, skilled nursing facilities) as long as appropriate monitoring and assessments follow. However, care teams with specialized training in DoC, experience in performing these trials, and providing subsequent assessments, titrations, and therapies are often required, making acute inpatient rehabilitation with a DoC program an ideal place for these managements. This consideration resonates with the recommendations in the AAN/ACRM/NIDILRR clinical practice guideline for DoC [27], as well as the minimum competency recommended for programs providing rehabilitation services to persons with DoC [28]. Several other considerations that may help conduct zolpidem and lorazepam trials are summarized below and may be areas for further studies.

One trial with an equivocal or a negative response does not denote that a patient would not benefit from these medications. Certain concomitant medical complications may affect the response. Repeated trials using the same agent, with or without dose titration, or at different times during daytime, should be considered in the following days. Periodically repeating the trials also needs to be considered to re-evaluate a patient’s response as neural recovery is a dynamic process and continues to evolve over time (e.g., neurotransmitter and receptor expression changes over time following traumatic brain injury [21]). The optimal re-trial interval is unknown. As shown in our cohort, it varied between around 30 days to 150 days. We would propose a re-trial interval of every 30–45 days using the same or a different agent. Further studies are needed.

Dose titration and timing need to be considered based on the patient’s performance to optimize the benefits. The responsiveness to zolpidem or lorazepam may change over time along recovery. Our results showed a significant proportion of the responders eventually did not require these medications to support their functionality they gained. Discontinuation of these medications needs to be considered when appropriate, especially when the hypnotic effect becomes more prominent [29]. Emerged patients may still benefit from these medications, especially those with severe functional impairments (sometimes, it may be related to conditions mimicking DoC, e.g., akinetic mutism [9], catatonia [13]).

With limited knowledge about the responders, current prospective studies failed to identify any potential phenotypical characteristics [11,17]. An alternative approach might be conducting a large prospective study with subgroup analysis on using zolpidem/lorazepam in the treatment of DoC. Pursuing randomized studies in a controlled environment after having a better understanding of certain phenotypical characteristics might be more yielding. Our results suggested the etiology (traumatic vs. anoxic) may serve as an identifier in future studies. Prioritizing the lorazepam trial in selected patients, especially those with suspected catatonic features, may be indicated in practice. As reported earlier [9,26], electrophysiological and neural connectivity parameters could be used to demonstrate the effects in a more objective and measurable manner.

### 4.2. Limitations

The study was conducted based on a chart review of clinical practice for patients with DoC. Management strategies and goals were aiming at the best possible patient care and functional outcomes. Several disadvantages of the study should be recognized. As a retrospective review study, the practice environment was uncontrolled. The results may potentially be confounded by practice preferences in the single facility, subjective bias (e.g., mostly open-labeled trials; determination of the positive trials based on narrative descriptions from the care team; no consistent objective measurements for the magnitude of response; however, this is also limited by reliable, objective evaluation tools in reality as even the CRS-R may miss significant behavioral evidence when they occur outside of the evaluation sessions or domains [30], as seen in Case 2, 8, 10), patient selection bias (e.g., choice of the trial regimen was made based on physicians’ impression of a patient’s condition), other medical comorbidities/complications (e.g., in Case 4, the undesirable response may be related to concomitant underlying infection), and other medication titrations (although efforts were made to minimize other medication changes, especially neurostimulants, during the days of the trials). These factors may either over- or underestimate the effectiveness of zolpidem and lorazepam. Natural recoveries and gains from therapies may also interplay, especially in those cases already at marginal emergence by the time of the first trial. Nevertheless, we believe the drastic behavioral/functional improvements shortly after administering zolpidem or lorazepam, rather than an expected hypnotic effect, were still able to justify the correlation of their therapeutic benefits for DoC. Advanced analysis was difficult due to the limited sample size and identifiable phenotypical characteristics of the responders. Because the wanted response is rare, an observatory cohort with a very large sample size may be needed to identify the characteristics.

## 5. Conclusions

Our results suggested the etiology of DoC, considering traumatic vs. anoxic injuries, may serve in guiding the clinical application of these medications in the treatment of DoC and in future prospective studies. We advocate for screening all patients with DoC using zolpidem and/or lorazepam.

## Figures and Tables

**Table 1:** Basic demographic information of the 146 patients.

		Full Cohort (*n* = 146)	Stroke (*n* = 11)	TBI (*n* = 87)	ABI * (*n* = 48)
Age at the time of injury (years; mean ± SD)		36 ± 15	44 ± 13	31 ± 14	41 ± 16
Sex (male; female)		108; 38	6; 5	69; 18	33; 15
Days since injury on admission (median (IQR))		62 (22–246)	78 (38–119)	60 (36–119)	73 (41–180)
Diagnosis on admission	UWS/VS	63	2	33	28
	MCS	74	7	48	19
	eMCS	9	2	6	1

* This included 4 patients who had a traumatic brain injury complicated with cardiac arrest and 2 patients who had a stroke complicated with cardiac arrest. As anoxic brain injury is usually more diffuse and profound, these patients were categorized into the anoxic brain injury group. TBI: traumatic brain injury; ABI: anoxic brain injury; UWS/VS: unresponsive wakefulness syndrome/vegetative state; MCS: minimally conscious state; eMCS: emerged from minimally conscious state; SD: standard deviation; IQR: interquartile range.

**Table 2:** The responders vs. the non-responders to the zolpidem and the lorazepam trials.

		TBI (*n* = 87)				ABI (*n* = 48)			
		Zolpidem Trial		Lorazepam Trial		Zolpidem Trial		Lorazepam Trial	
Trial rate *		56.3% (49/87)		33.3% (29/87)		62.5% (30/48)		29.2% (14/48)	
Positive rate **		10.2% (5/49)		6.9% (2/29)		0.0% (0/30)		28.6% (4/14)	
		R (*n* = 5)	NR (*n* = 44)	R (*n* = 2)	NR (*n* = 27)	R (*n* = 0)	NR (*n* = 30)	R (*n* = 4)	NR (*n* = 10)
Age at the time of injury (years; mean ± SD)		26 ± 12	34 ± 14	22 ± 3	33 ± 14	/	41 ± 15	37 ± 11	48 ± 14
Sex (male; female)		5; 0	35; 9	2; 0	24; 3	/	23; 7	3; 1	7; 3
Days since injury on admission (median (IQR))		62 (32–81)	64 (45–177)	42 and 113	76 (49–266)	/	88 (50–202)	48 (31–78)	55 (39–106)
Diagnosis on admission	UWS/VS	2	22	0	9	/	20 10	1 3	5 5
	MCS	3	22	2	18				

* This presents the number of patients who underwent medication trials in each group. ** This presents the number of positive trials (i.e., responders) among the medication trials in each group. TBI: traumatic brain injury; ABI: anoxic brain injury; R: responders; NR: non-responders; UWS/VS: unresponsive wakefulness syndrome/vegetative state; MCS: minimally conscious state; eMCS: emerged from minimally conscious state; SD: standard deviation; IQR: interquartile range.

**Table 3:** The zolpidem responders’ demographics, trial information, and outcomes.

Case	Sex	Age	Etiology	Trial Time (Days Since Injury) and Regimen	Amantadine	Diagnosis (CRS-R Total (Subscales *))		Lorazepam Trial
						Before Trial	After Trial	
1	Male	28	TBI	D56-D60, 10 mg QD D61-D72, 10 mg BID D73-D80, 10 mg TID D81-D82, 10 mg QD D83-D90, 10 mg BID D91-D99, 15 mg BID	D49-D55, 150 mg BID D56-D60, 150 mg BID D61-D72, 150 mg BID D73-D80, 150 mg BID D81-D82, 150 mg BID D83-D89, 150 mg BID D90-D99, 50 mg BID	MCS D42, 7 (004102) D49, 8 (004112)	eMCS D56, 11 (004133) D63, 15 (106233) D70, 11 (006113) D77, 23 (256433) D82, 15 (134133) D91, 15 (105432) D99, 22 (255433) <no CRS-R was administered further>	No
	Behavioral changes/functional improvements: Verbalization. D56: “Patient had been administered 10 mg zolpidem at 11:36 a.m. When we entered the room (1:05 p.m.) his aunt reported that patient spoke the word “fart” after having done so. This is the first intelligible verbalization of which we are aware.” “Patient was attempting to verbalize frequently and produced several intelligible responses, including answering “Fine” to “How are you?”, stating “cops” and “right here.” Other attempts at verbalization were produced in a whisper and were difficult to understand.” Discontinuation process: Discharged on D99 on zolpidem; had been discontinued by the time of the first clinic follow-up half a year later. Long-term outcome: A total of 4 years post-injury challenge program discharge note: “He is able to ambulate without a device and performs his own ADL activities with supervision. He displays poor initiation but will complete light iADL activities with supervision. He has begun to participate in the management of his own medications.” He also applied to the Texas Workforce Commission for supported employment.							
2	Male	18	TBI	D600, 5 mg ONCE (equivocal) D606-D607, 10 mg QD D611-D617, 10 mg QD D618-D633, 5 mg QD	D593-D599, 100 mg BID D600-D606, 100 mg BID D607-D633, 150 mg BID	MCS D586, 15 (152322) D591, 13 (042322)	eMCS D607, patient was considered clinically eMCS with functional communication. <no CRS-R was administered further>	No
	Behavioral changes/functional improvements: More accurate on IQBA at the evaluation conducted 45 min after taking zolpidem. D606: “Patient underwent zolpidem trial at 12:45 p.m. this afternoon. He initially visually tracked to the NP fellow as she walked to the left, which represents an improvement. However, arousal was variable.” “The IQBA was administered to assess yes/no accuracy of his RUE finger movements. He was able to answer 3 questions, with all 3 being correct. This does not represent a change from previous IQBA administrations.” D607: “Zolpidem administered at 12:30 p.m. At 1:15 p.m., total responses 8/8 questions, accurate 4/8 questions, response time ~10 s. At 2:10 p.m., total responses 6/8 questions, accurate 6/6 questions, response time ~10 s. At 4:00 p.m., total response 2/2 questions, accurate 2/2 questions. Patient signed a need to stop using thumb and vocalization, so evaluation was discontinued.” Discontinuation process: Re-scheduled to bedtime on D634 to see if it affected sleep; discontinued on D641 without documented reason. Long-term outcome: Not available; lost to follow-up.							
3	Male	16	TBI	D46, 5 mg ONCE D47, 7.5 mg ONCE D48-D51, 5 mg QD D52-D136, 5 mg BID	D39-D45, 150 mg BID D46-D47, 150 mg BID D48-D83, 150 mg BID D84-D136, 200 mg BID	eMCS D30, 9 (032112) D32, 10 (032311) D37, 13 (232312) D40, 18 (246312)	eMCS <no CRS-R was administered further>	Equivocal D56 1 mg D58 1 mg
	Behavioral changes/functional improvements: Increased arousal, attention, processing speed. D46: “Patient had received a dose of 5 mg of zolpidem at 1:10 p.m. (Seen at 2:30 p.m.) Patient appeared wide awake.” “Patient with quicker response times with regard to left arm movement.” “Patient able to answer several yes/no questions with his left hand with good accuracy.” “Patient was able to read single words and point to the words commanded with 100% accuracy.” “It should be noted that reaction and response time was noted to be improved compared to previous days.” Discontinuation process: Discharged on D136 on zolpidem; had been discontinued by the time of the first clinic follow-up 2 months later. Long-term outcome: A total of 7 months post-injury: “Upon discharge, he was able to ambulate 150 ft with assistance, Maximum to Total Assist for most ADLs, Modified Independence for comprehension, Standby for expression, Moderate Assist for memory. He’s currently at Challenge Program. School is sending regular curriculum content to his home computer. He’s doing well.”							
4	Male	46	TBI	D144, 5 mg QD (no reports regarding responses) D151-D158, 10 mg QD	D137-D142, 100 mg QD D143-D147, 100 mg BID D148-D158, 200 mg BID	UWS/VS D134, 5 (002111) D140, 5 (002111)	UWS/VS D151, 7 (001321) (D158, transferred due to medical deterioration)	No
	Behavioral changes/functional improvements: Reproducible movement to command (during CRS-R). D151: “Patient received medication trial (zolpidem) prior to CRS-R testing (at 9:00 a.m.).” During CRS-R (at 10:00 a.m.), “patient demonstrated increased frequency, type and range of movements.” “Patient responded correctly in ¾ trials (reproducible movement to command with ‘look at the ball’ and ‘look at the cup’), vocalized x3.” D154-D155: Patient has been having fevers. D156: “Patient demonstrated increased oral facial movements (labial spread, buccal tension and elevation, mouth opening, lip pucker, and bilateral eyebrow elevation) and body movements (thoracic positioning/posturing), initiated lingual protrusion in 15/25 given mod max cues.” Discontinuation process: Discharged on D158 on zolpidem; discontinued at outside hospital. Long-term outcome: A total of 5 months post-injury, patient developed pneumonia but recovered. He resided in a nursing home. A total of 7 months post-injury, he was admitted for pneumonia; there was concern for shunt malfunction; after goals of care discussion, family elected not to proceed surgical treatment, elected to continue management of seizure, pneumonia, and pain; family was not ready for hospice, but aware it as an option.							
5	Male	23	TBI	D182, 5 mg ONCE D186, 7.5 mg ONCE D187-D195, 7.5 mg BID D196-D207, 10 mg-5 mg BID (higher PM dose made him sleepy) D208-D210, 15 mg-5 mg BID	D175-D181, 100 mg BID D182-D186, 100 mg BID D187-D195, 100 mg BID D196-D207, 100 mg BID D208-D210, 100 mg BID	MCS, but close to eMCS D131, 14 (152321) D138, 16 (152422) D146, 11 (122321) D152, 16 (152422) D165, 17 (152423)	eMCS D187, 21 (256323) <no CRS-R was administered further>	No
	Behavioral changes/functional improvements: More responsive and interactive. D182: “Patient observed with zolpidem trial (administered at 9:50 a.m.). Patient more responsive than last few days and interactive (at 10:30 a.m.). Patient positively endorsed feeling tired.” “Patient reported to be willing to attempt a second zolpidem trial. Patient’s performance improved today while under the effects of zolpidem.” D187: “Patient received 7.5 mg Zolpidem at approximately 7:30 a.m. Patient scored CRS-R 21 overall (performed at 9:20 a.m.), demonstrating both functional object use and functional communication.” Discontinuation process: Discharged on D210 on zolpidem; discontinued months after discharge. Long-term outcome: A total of 6 years post-injury, he remains total assist in ADL and mobility, non-verbal, answering questions by eye movements (vertical for yes, horizontal for no); status post intrathecal baclofen pump placement, receiving periodic botulinum toxin injections. Continues to reside in a skilled nursing facility.							

* The subscales are presented as Communication, Visual function, Motor function, Auditory function, Oromotor/verbal function, Arousal (achieving C2 and/or M6 indicates eMCS; achieving either C1, or V2, or M3, or A3, or O3 indicates MCS; otherwise, UWS/VS). TBI: traumatic brain injury; ABI: anoxic brain injury; UWS/VS: unresponsive wakefulness syndrome/vegetative state; MCS: minimally conscious state; eMCS: emerged from minimally conscious state; QD: once daily; BID: twice per day in the early morning and at noon; TID: three times per day; CRS-R: Coma Recovery Scale-Revised; IQBA: Individualized Quantitative Behavioral Assessment; ADL: activities of daily living; iADL: instrumental activities of daily living; D: days since onset of brain injury.

**Table 4:** The lorazepam responders’ demographics, trial information, and outcomes.

Case	Sex	Age	Etiology	Trial Time (Days Since Injury) and Regimen	Amantadine	Diagnosis (CRS-R Total (Subscales *))		Zolpidem Trial
						Before Trial	After Trial	
6	Male	48	ABI	D217, 1 mg ONCE D223, 2 mg ONCE D224-D229, 2 mg TID D230-D231, 2.5 mg TID D232-D233, 3 mg TID D234-D241, 4 mg TID	D210-D216, 100 mg BID D217-D241, 100 mg BID * weaning off of clonazepam (for seizure and spasticity) during the process, discontinued on D238	MCS D214, 9 (003231)	MCS D223, 12 (142122) D229, 11 (113231) D237, 12 (033231) <no CRS-R was administered further>	Negative D86 10 mg D219 10 mg
	Behavioral changes/functional improvements: Increased arousal and verbalization. D217: “Lorazepam 1 mg IV trial (at 8:34 a.m.) resulted in an arousal for a short period of time.” “Patient speaking without speaking valve. Able to count 1-10. Repeated words with ~25% accuracy (at 11 a.m.).” D223: “(Lorazepam administered at 8:00 a.m.; patient seen by speech therapist at 11 a.m.) Increased verbalization was noted by family and therapists.” D224: Lorazepam was administered at 7:00 a.m., 1:00 p.m., 7:00 p.m. “Patient produced single words using pacing with mod cues (at 10:00 a.m.).” “Patient kicked a ball x2 trials although response required ~20 s (around 1.5 h after lorazepam was given).” Discontinuation process: Continues to take TID with varying dosage over the years; weaned down to 1 mg due to arousal. Long-term outcome: A total of 4 years post-injury: Total assist for ADLs and mobility; metal trach for secretion, requiring frequent suctioning; also taking baclofen and clonazepam for tone, which was worsening over time. He had been transitioned into a hospice program 3 years post-injury. A total of 6 years post-injury: He visited the emergency room for PEG dislodge and had replacement.							
7	Male	22	ABI	D47, 1 mg ONCE D48, 1 mg ONCE D77-D85, 2 mg QD	D40-D46, 100 mg BID D47-D85, 100 mg BID	MCS/eMCS D29, 6 (012111) D33, 8 (021221) D36, 8 (031211) D41, 15 (046212) D44, 8 (032111)	eMCS D50, 14 (135311) D54, 13 (142312) D58, 17 (146222) <no CRS-R was administered further>	Equivocal D54 5 mg D58 10 mg D73 10 mg
	Behavioral changes/functional improvements: Increased cognitive ability. D47: “(Lorazepam was given at 1:00 p.m.; patient was seen by speech therapist at 3:30 p.m.) Patient observed to answer yes/no questions at 75%.” (D42-D43: “Ambiguous head nods for yes, shakes for no to egocentric questions. Ambiguous command following. Attention is a barrier.”) D77: “Positive response to lorazepam.” (No further details could be obtained). D78: “Patient ambulated greater than 100’ with therapist walking to the left of patient.” “Throughout ambulation, patient with no episodes of hyperventilation and crying (which had been an issue before). It was noted that the patient responded 100% of the time with use of a YES/NO board. This will be implemented until patient begins to verbalizing more regularly and reliably. Patient with second dose of lorazepam (2 mg today).” Discontinuation process: Discharged on D85 on lorazepam; started weaning 10 months post-injury as patient continued to improve; no issue with arousal; off of lorazepam by 13 months post-injury. Long-term outcome: A total of 2 years post-injury: Patient had a baby; passed driving test; worked in construction but remained on disability. Still has anger/irritability issues but under control.							
8	Male	24	TBI	D76, 1 mg ONCE D79, 2 mg ONCE D86-D87, 0.5 mg QD	None	MCS D51, 11 (035111) D58, 12 (035112) D66, 11 (035111) D69, 14 (045122) D73, 11 (035111)	eMCS D79, 14 (035222) D87, 14 (045122) (demonstrated functional object use with both a cup and a pen, however, his attention limits his performance of these behaviors on 4/4 trials) <Patient was considered clinically eMCS; no CRS-R was administered further>	No
	Behavioral changes/functional improvements: Less restless; more attentive and engaged with environment. D76: “(Lorazepam was administered at 10:30 a.m.; patient was seen by neuropsychologist at 2:30 p.m.) Overall less restlessness but more agitation.” D79: “(Lorazepam was administered at 7:57 a.m.; CRS-R was performed at 10:30 a.m.) patient responded to paired egocentric yes/no questions via head nod/shake during PT session. Patient with increased unintelligible verbalization attempts with and without phonation outside of this assessment.” D87: “Lorazepam was given just before the session. Once in the gym, he played wii baseball with occupational therapy. Patient demonstrated appropriate response to Pop-A-Shot basketball. He took the ball from the receptacle and appropriately rotated hand into basketball shooting position; however, dropped the ball each time. Of note, he was observed squeezing legs together in response to losing control of the basketball. On one occasion he caught dropped ball with legs and grabbed it again with left hand.” Discontinuation process: It appears patient improved rapidly during the period of time having lorazepam trials with different dosages. He was later considered emerged and started on Ritalin. Long-term outcome: A total of 1 year post-injury, the patient was able to feed and groom himself, dressing upper and lower under supervision, maximum assist in bowel/bladder/bath care, supervision/contact guard assist in basic transfer. He was able to walk 40 ft with minimum assist, using a wheelchair for mobility. A total of 2.5 years post-injury, he continued to reside in a long-term care facility and continued to have short-term and long-term memory dysfunction.							
9	Female	36	ABI	D96, 1 mg ONCE D97, 2 mg ONCE D99, 1 mg TID D100-D103, 1 mg QD D104-D112, 2 mg QOD D113-D116, 1 mg QD D117-D123 held for washout D124-D130, 1 mg TID D131-D132, 1.5 mg TID D133-D137, 0.5 mg TID D138-D140, 1 mg TID D141-D143, 1 mg QID D144-D145, 1.5 mg QID D146-D149, 2 mg QID D150-D160, 2.5 mg QID	D89-D95, 100 mg BID D96-D109, 100 mg BID Discontinued on D110	eMCS D33, 6 (012111) D38, 13 (015322) D48, 18 (156321) D52, 18 (155322) D55, 20 (156323) D63, 12 (042321) D66, 17 (145322) (Patient was able to functionally communicate but is often distracted and inattentive.)	eMCS <no CRS-R was administered further>	No
	Behavioral changes/functional improvements: Increased initiation, attention, verbalization, processing time; positive affect; had her best participation in therapy. D96: “Patient received 1mg IV lorazepam at 2:00 p.m. (Seen by neuropsychologist and speech therapist at 2:15 p.m.) Patient participated in speech therapy with increased production of speech, improved intelligibility, increased phrase length and accurate responses to phrase completions and identifications of objects. Patient had increased spontaneous verbalizations, initiating communication of wants and needs. Patient sang and moved her upper extremity. Patient recalled therapists’ names and disciplines with cuing. Patient observed to independently initiate 5-word sentences such as ‘I want something to drink’ during the session.” Discontinuation process: Discharged on D160 on lorazepam to a post-acute facility; lost to follow-up in our system. Long-term outcome: Not available; lost to follow-up.							
10	Male	20	TBI	D296-D300, 1 mg QD D301-D314, 2 mg QD D315-D321, 2 mg-1 mg BID D322-D347, 2 mg BID D348-D379, 1 mg BID	Re-admitted on D295 on 100 mg BID D296-D339, 100 mg BID D340-D379, 150 mg BID	eMCS D147, 15 (045321) D152, 14 (035321) D155, 18 (155421) (Patient demonstrated eMCS by answering >10 egocentric yes/no questions accurately once test was completed.) D158, 13 (035311) D161, 16 (145321) D165, 17 (236321)	eMCS <no CRS-R was administered further>	Negative D133 5 mg D137 10 mg D154 5 mg D349 5 mg
	Behavioral changes/functional improvements: This initially occurred at outside hospital; per family, it was helpful, thus continued. Patient continued to participate well in therapy. Discontinuation process: Continues to take lorazepam 2 mg-1 mg BID 2 years post-injury. Long-term outcome: A total of 3 years post-injury, patient has completed 3 phases inpatient rehabilitation and Rehab Without Walls; continued to require maximum to total assist in functional mobility; continued to have poor initiation, verbalization but able to answer yes/no questions via head nods/shakes; pending intrathecal baclofen pump placement after a positive trial for spasticity management; continued outpatient therapy; lived at home with family.							
11	Male	41	TBI&ABI	D136, 1 mg ONCE (negative) D321, 1 mg ONCE D323–324, 1 mg QD D325-D327, 2 mg QD D331-D337, 1 mg QD D338, 2 mg QD D339, 2 mg BID + 2 mg ONCE D340, 3 mg BID D341, 4 mg BID D342-D359, 3 mg BID D390, 2 mg ONCE D393-D394, 3 mg QD D395-D401, 3 mg BID D402-D420, 2 mg BID D421-D430, 2 mg-1 mg BID D431-D433, 1.5 mg-1 mg BID D434-D449, 2 mg-1 mg BID	D314-D320, 200 mg BID D321-D359, 200 mg BID D390-D449, 200 mg BID (D360: Transferred out for scheduled surgery; while at OSH, lorazepam was tapered to stop by Neurology, stating “lorazepam is a CNS depressant and a stimulant effect is pathophysiologically unlikely.”)	eMCS D124, 12 (142311) D128, 13 (151312) D131, 16 (252412) D134, 16 (252412) D137, 16 (252412) D141, 15 (152322)	eMCS <no CRS-R was administered further>	Equivocal D131 5 mgD133 10 mg
	Behavioral changes/functional improvements: Increased alertness, spontaneous movement, and affect. D321: “Patient participating in lorazepam trial (given 10:15 a.m.). (Seen by speech therapist at 11:00 a.m.) Patient noted to be more alert with increased spontaneous movement and affect. Patient noted with increased bilabial movement and left lip retraction. Patient followed simple 1-step directions to turn head to the left/right and bring head forward given multiple repetitions and min to mod verbal and visual cues. Movements noted to be larger than typically seen.” D323: “Increased responsiveness to yes/no questions via head movement. Patient responded to approximately 80% of yes/no questions targeting orientation and delayed recall of information.” Discontinuation process: Started weaning due to cognitive alteration and improvements seen after stopping lorazepam prior to discharge on D449. The patient was not taking lorazepam in the following admissions. Long-term outcome: A total of 3 years post-injury, the patient remained total assist in all ADLs and for all mobility; lived with parents.							

* The subscales are presented as Communication, Visual function, Motor function, Auditory function, Oromotor/verbal function, Arousal (achieving C2 and/or M6 indicates eMCS; achieving either C1, or V2, or M3, or A3, or O3 indicates MCS; otherwise, UWS/VS). TBI: traumatic brain injury; ABI: anoxic brain injury; UWS/VS: unresponsive wakefulness syndrome/vegetative state; MCS: minimally conscious state; eMCS: emerged from minimally conscious state; QD: once daily; BID: twice per day in the early morning and at noon; TID: three times per day; QID: four times per day; CRS-R: Coma Recovery Scale-Revised; IQBA: Individualized Quantitative Behavioral Assessment; ADL: activities of daily living; D: days since onset of brain injury.

## Data Availability

Anonymized data can be made available upon reasonable request.
